# Stress Marker Signatures in Lesion Mimic Single and Double Mutants Identify a Crucial Leaf Age-Dependent Salicylic Acid Related Defense Signal

**DOI:** 10.1371/journal.pone.0170532

**Published:** 2017-01-20

**Authors:** Eve Kaurilind, Mikael Brosché

**Affiliations:** 1 Institute of Ecology and Earth Sciences, University of Tartu, Tartu, Estonia; 2 Institute of Agricultural and Environmental Sciences, Estonian University of Life Sciences, Tartu, Estonia; 3 Division of Plant Biology, Viikki Plant Science Centre, Department of Biosciences, University of Helsinki, Helsinki, Finland; 4 Institute of Technology, University of Tartu, Tartu, Estonia; Beijing Forestry University, CHINA

## Abstract

Plants are exposed to abiotic and biotic stress conditions throughout their lifespans that activates various defense programs. Programmed cell death (PCD) is an extreme defense strategy the plant uses to manage unfavorable environments as well as during developmentally induced senescence. Here we investigated the role of leaf age on the regulation of defense gene expression in *Arabidopsis thaliana*. Two lesion mimic mutants with misregulated cell death, *catalase2* (*cat2*) and *defense no death1* (*dnd1*) were used together with several double mutants to dissect signaling pathways regulating defense gene expression associated with cell death and leaf age. PCD marker genes showed leaf age dependent expression, with the highest expression in old leaves. The salicylic acid (SA) biosynthesis mutant *salicylic acid induction deficient2* (*sid2*) had reduced expression of PCD marker genes in the *cat2 sid2* double mutant demonstrating the importance of SA biosynthesis in regulation of defense gene expression. While the auxin- and jasmonic acid (JA)- insensitive *auxin resistant1* (*axr1*) double mutant *cat2 axr1* also led to decreased expression of PCD markers; the expression of several marker genes for SA signaling (*ISOCHORISMATE SYNTHASE 1*, *PR1* and *PR2*) were additionally decreased in *cat2 axr1* compared to *cat2*. The reduced expression of these SA markers genes in *cat2 axr1* implicates AXR1 as a regulator of SA signaling in addition to its known role in auxin and JA signaling. Overall, the current study reinforces the important role of SA signaling in regulation of leaf age-related transcript signatures.

## Introduction

Plants are sessile organisms and typically experience altered environmental conditions throughout their life cycle. Plant survival depends on their ability to acclimate to the surrounding environment and requires systemic signaling from mature to young developing leaves [1–4]. Reactive oxygen species (ROS) are produced during cell metabolism and production rates increase under stress conditions leading to plant damage [5,6]. However, ROS are not only damaging agents, they are actively produced by the plant and used as signaling molecules both in development and in response to abiotic and biotic stress [7–9]. Hydrogen peroxide (H_2_O_2_) is the most stable ROS and an important signaling molecule involved in triggering tolerance to various abiotic and biotic stresses at low concentrations; high concentrations lead directly to programmed cell death (PCD) [10]. The life-time of ROS signals is controlled by antioxidants and ROS scavenging enzymes. About 70% of H_2_O_2_ is produced during photorespiration [11] which may help protect the cell and provide adaption to unfavorable conditions [5]. Catalases are the main enzymes detoxifying H_2_O_2_ to H_2_O and O_2_ in the peroxisome [12]. However, catalases can also be involved in the removal of H_2_O_2_ from other subcellular compartments and thus function as a sink for cellular H_2_O_2_ [13].

Plant responses to the environment also needs to be integrated with growth and developmental processes. Since activation of defenses against stress is energetically costly, plants need to optimize between growth and defense strategies. Consequently, suboptimal growth conditions typically cause an altered plant phenotype. The stress-induced morphogenic response (SIMR) has been proposed as a concept explaining how stress leads to altered growth, and is regulated by auxin, ROS and antioxidants [14–16]. In addition to SIMR, plants also have several other long distance signaling responses, where a signal initiated in a local tissue spreads to distal tissues. These include systemic acquired resistance (SAR; [17]), induced systemic resistance (ISR, [18]) and systemic acquired acclimation (SAA; [19].) SAR has been extensively characterized in relation to pathogen infection, and execution of SAR requires the hormone salicylic acid (SA) and various other signaling molecules including ROS, azelaic acid, pipecolic acid and the co-transcriptional regulator NONEXPRESSER OF PR GENES 1 (NPR1) [17]. ISR is initiated after infection of roots by nonpathogenic microbes, which induce a resistance response in distal leaves. ISR does not require SA, but rely on the hormones ethylene, jasmonic acid (JA) and NPR1 [18]. The SAA response to various abiotic stresses depends on ROS, Ca^2+^ signaling and abscisic acid (ABA) [19]. Furthermore, other plant hormones including auxin and cytokinins (CK) are involved in long distance signaling [20,21]. However, several questions remain to be answered on how different plant hormones together with ROS and transcriptional re-programming regulate the complex interactions between development and stress responses.

*Arabidopsis thaliana* mutants with misregulated cell death (also known as lesion mimic mutants, LMMs) have long been used to identify regulators of defense responses and PCD [22,23]. The Arabidopsis *cat2* mutant, deficient in the peroxisomal ROS scavenger *CATALASE2*, develops lesions that are day length and light intensity dependent [12]. Identification of positive and negative regulators of PCD in *cat2* indicate that several signaling pathways are activated in parallel and influence the timing and extent of PCD [24]. These regulators include SA and AUXIN RESISTANCE 1 (AXR1) [24]. AXR1 regulates the activity of Skp-Cullin-F-box (SCF) complexes involved in protein degradation [25]. The JA receptor COI1 and the auxin receptor TIR1 are regulated through this mechanism and the *axr1* mutant is both auxin and JA insensitive. The LMM *dnd1* (*defense no death1*) displays a growth dependent lesion phenotype [26,27]. *DND1* encodes CYCLIC NUCLEOTIDE GATED CHANNEL 2, which link Ca^2+^ transport to downstream defense signaling [28]. Like many other LMMs, cell death in *dnd1* is regulated through SA signaling [27].

While young leaves of *cat2* have conditional day-length dependent lesions, cell death is prevented in newly developed leaves of mature plants in *cat2* [24]. Furthermore, when *cat2* plants grown in high CO_2_ concentration (which suppress cell death) are transferred to ambient air and high light treated, cell death is extensive in old but not young leaves [29]. This indicates the presence of a signal originating in old leaves that increases the viability of newly developed leaves, or some other mechanism that protects young leaves. Cell death in *cat2* and a second LMM *dnd1* (*defense no death1*) is reduced in double mutants with *sid2* that reduce the amount of SA [24,27]. Furthermore, loss of function mutants in SA biosynthesis or signaling leads to delayed senescence [30]. Thus, several lines of evidence suggest that one or more signals, including SA, move from old leaves to young leaves when plants are constitutively exposed to oxidative stress such as in LMMs. However, the mechanisms of oxidative stress development and the identity of signals from old to young leaves is unclear.

**Fig 1:**
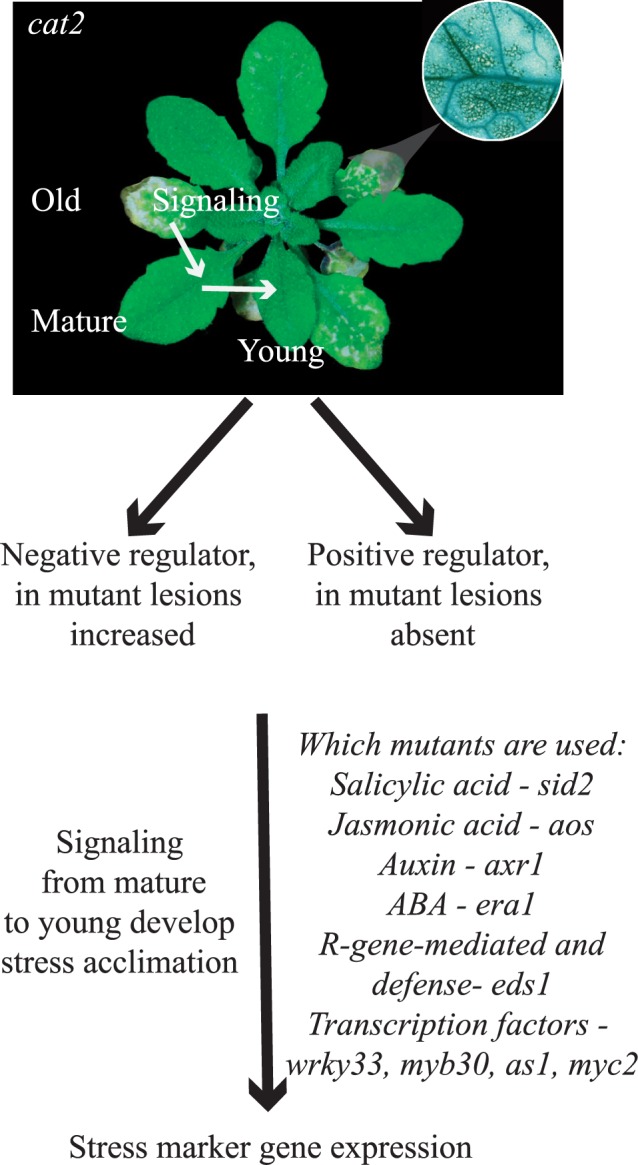
Research scheme for plant signaling from mature to young leaves. Lesion formation in *cat2* typically starts from older leaves. To dissect the role of leaf age on PCD regulation, leaves were separated into the classes old (with visual lesions), mature (fully developed without lesions) and young (developing). Several positive and negative regulators of *cat2* cell death were previously identified ([24]; see also Table 1), and the most informative of these were used to gain insights into the regulation of expression of cell death marker genes.

To dissect possible defense signals and their regulators during plant growth, we took advantage of our previously established collection of *cat2* double and triple mutants [24] and similar mutants in a second LMM *dnd1* [27]. We selected mutants where cell death in *cat2* and *dnd1* was reduced and performed gene expression analysis in leaves at different developmental stages defined here as young, mature and old (Fig 1). This study reveals that both auxin and SA signaling are important regulators of defense gene expression in leaves of different age classes.

## Materials and Methods

### Plant Materials and Growth Conditions

All the mutants were in the Columbia-0 (Col-0) background and Col-0 was the control for all experiments. Double and triple mutants were in the *cat2* (SALK_076998) background as previously described [24]. The *dnd1* double mutants were previously described [27].

Sterilized seeds were placed on Murashige and Skoog (MS) medium (1/2 MS salts, and 0.7% agar), stratified for three days and transferred to a growth chamber (Sanio Electric Co) at 21°C/19°C under a 12 h day/12 h night regime, light intensity 120 μmol m^-2^ s^-1^, and 70% relative humidity. One week old plants were transplanted into pots with 1:1 peat:vermiculate and grown on soil for four weeks in controlled growth rooms. Three experimental repeats were used for gene expression analysis.

Plant material was collected from five weeks old plants. Three groups of leaves at different developmental stages were selected based on leaf age: old (leaves with visual lesions, leaf position 5–7), mature (fully developed leaves without lesions, leaf position 9–12), young (developing leaves, no lesions, leaf position 12–14). Eight leaves from each age class were pooled, frozen in liquid nitrogen and stored at -80°C. Total RNA was extracted using the Spectrum Total RNA extraction kit (Sigma Aldrich).

### qPCR Gene Expression Analyses

The expression of marker genes involved in PCD regulation was measured with real time quantitative PCR (qPCR). Three biological repeats were used for gene expression analysis with qPCR. RNA was treated with DNAseI and reverse transcription was performed using 2 μg of RNA with the RevertAid Premium Reverse Transcriptase (RT) and Ribolock Rnase inhibitor according to manufacturer’s instructions (Thermo Fisher Scientific). After reverse transcription the reaction was diluted to the final volume of 100 μl. 1 μl was used for PCR with EvaGreen ROX (Solis Biodyne). The cycle conditions in the ABI 7900HT Fast RT PCR System (Applied Biosystems) were: 95°C 10 min, 40 cycles with 95°C 15 s, 60°C 30 s, 72°C 30 s and ending with melting curve analysis. Normalization of the data was performed in qBase 2.0 (Biogazelle), with three reference genes *TIP41*, *YLS8* and *SAND*. Primer amplification efficiencies were determined in qBase from a cDNA dilution series. Primer sequences and amplification efficiencies can be found in S1 Table. Data normality was tested and subsequently 2-base logarithmed for statistical analyses. Factorial ANOVA posthoc analyses Fisher LSD was used to evaluate significant differences between mutant and leaf age, and One-Way ANOVA to changes in gene expression with leaf age (Statistica 7.1, Stat Soft Inc).

### Selection of Marker Genes

Previous analysis of gene expression in *cat2* using genes from the gene ontology category “cell death” in [24] identified several genes with strongly increased expression in various LMMs, and in response to SA and pathogen treatment. 26 genes of these genes were tested at different leaf age classes (old, mature, young) in Col-0 and *cat2*. Five marker genes; *FMO1*, *PLA2A*, *WRKY75*, *WRKY40* and *GLTP* were chosen as qPCR marker genes based on significant differences in expression between different leaf ages.

### Analysis of Marker Gene Expression in Public Gene Expression Data

The selected marker genes used for qPCR were analyzed with the Condition Search tool “Perturbations”in Genevestigator https://genevestigator.com/gv/doc/intro_plant.jsp [31]. Marker gene expression is shown in response to hormone and pathogen treatment and in LMMs.

## Results

### Identification of Suitable Marker Genes to Study Cell Death

**Fig 2:**
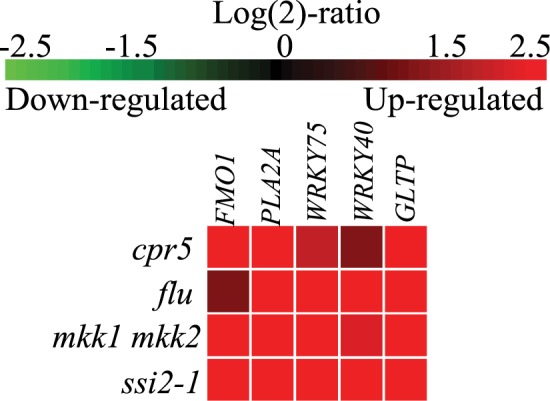
Expression of PCD marker genes in LMMs *cpr5*, *flu*, *mkk1 mkk2* and *ssi2-1*. Expression of *FMO1*, *PLA2A*, *WRKY75*, *WRKY40* and *GLTP* as visualized by investigating selected mutants in Genevestigator using the Perturbations tool. Green indicates decreased expression and red increased expression.

Multiple signals are involved in plant systemic signaling in relation to biotic and abiotic stress and also during leaf ageing [1–3,19,32]. To find marker genes that would be informative for stress responses associated with ageing in *cat2*, we took advantage of our previous analysis of several independent *cat2* gene expression experiments and other LMMs (Fig 1; Table 1, [24]). From these experiments, we selected five genes associated with the gene ontology category cell death: *FLAVIN-DEPENDENT MONOOXYGENASE 1 (FMO1)*, *PHOSPHOLIPASE A2A (PLA2A)*, *WRKY DNA-BINDING PROTEIN 75 (WRKY75)*, *WRKY DNA-BINDING PROTEIN 40 (WRKY40)* and *GLYCOLIPID TRANSFER PROTEIN (AT4G39670*, hereafter *GLTP)*. Analyses of LMM gene expression profiles in Genevestigator shows that all five stress marker genes had high expression in: *ssi2-1 (suppressor of SA-insensitive 2)*, *mkk1 mkk2 (mitogen activated protein kinase kinase 1 and 2)* and *cpr5 (constitutive expression of pr genes 5)*. Furthermore, high expression was also seen in *flu (fluorescent in blue light)* that develops cell death after accumulation of singlet oxygen (Fig 2).

**Table 1:** Summary of *cat2* double mutant cell death phenotypes. The *cat2* single mutant develops extensive cell death when grown on soil in 12/12h light dark conditions [24]. The double mutants were used for gene expression analysis to determine potential signaling pathways involved in gene regulation during PCD.

**Mutant**	**Extent of cell death (Kaurilind et al., 2015)**	**Possible mechanism involved**	**Reference to single mutant**
*cat2 sid2*	Very few lesions	SA biosynthesis	[33]
*cat2 eds1*	Very few lesions	Defense regulation in SA pathway	[34]
*cat2 aos*	Lesions	JA biosynthesis	[35]
*cat2 era1*	No lesions	Protein farnesylation, ABA hypersensitive	[36]
*cat2 axr1*	No lesions	Regulation of SCF complex activity JA and auxin-dependently	[25]
*cat2 as1*	No lesions	TF, JA and pathogen responses	[37]
*cat2 myb30*	Few lesions	TF, cell death regulation	[38]
*cat2 myc2*	Few lesions	TF, JA/ABA crosstalk	[39]
*cat2 wrky33*	Few lesions	TF, pathogen responses	[40]

**Fig 3:**
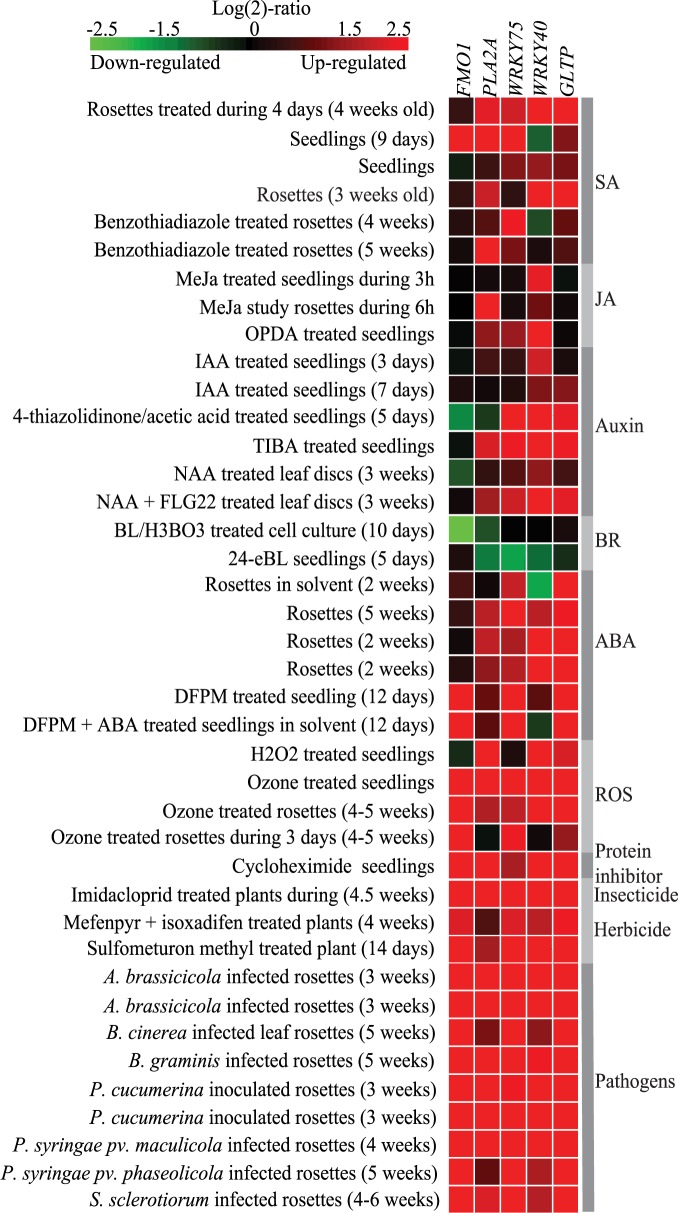
PCD marker gene expression related to hormone, ROS and pathogen response. Expression of *FMO1*, *PLA2A*, *WRKY75*, *WRKY40* and *GLTP* as visualized by investigating selected hormone, pathogen and ROS treatments in Genevestigator using the Perturbations tool. Green indicates decreased expression and red increased expression. Plant age is indicated in parenthesis.

Ideally, the marker genes should reflect the output of independent signaling pathways in addition to cell death, for example differential regulation by separate hormones. To check for other treatments that regulate expression of the five selected markers we used Genevestigator (Fig 3). As expected from genes associated with cell death, all five genes had increased expression in response to pathogen infection. Similarly, different ROS treatments (ozone and H_2_O_2_) increased expression of *FMO1*, *PLA2A*, *WRKY75*, *WRKY40* and *GLTP*. Subtle differences were found in response to different hormones, where expression of *FMO1* was mostly hormone independent. *PLA2A* expression increased by most hormone treatments except for brassinosteroid applications, which led to decreased expression (Fig 3). *WRKY75* expression increased in response to SA, auxin and ABA, while *WRKY40* expression increased by all hormones except the brassinosteroid treatment. Finally, *GLTP* expression increased by application of SA, auxin and ABA, but not JA or brassinosteroids. Thus, although the selected marker genes all reflect ROS, pathogen infection and cell death signaling, they may also be associated with distinct combinations of hormone signaling pathways. Especially *FMO1* expression represent a more specific (mostly hormone independent) signaling context.

### Expression of Marker Genes in Single Mutants

**Fig 4:**
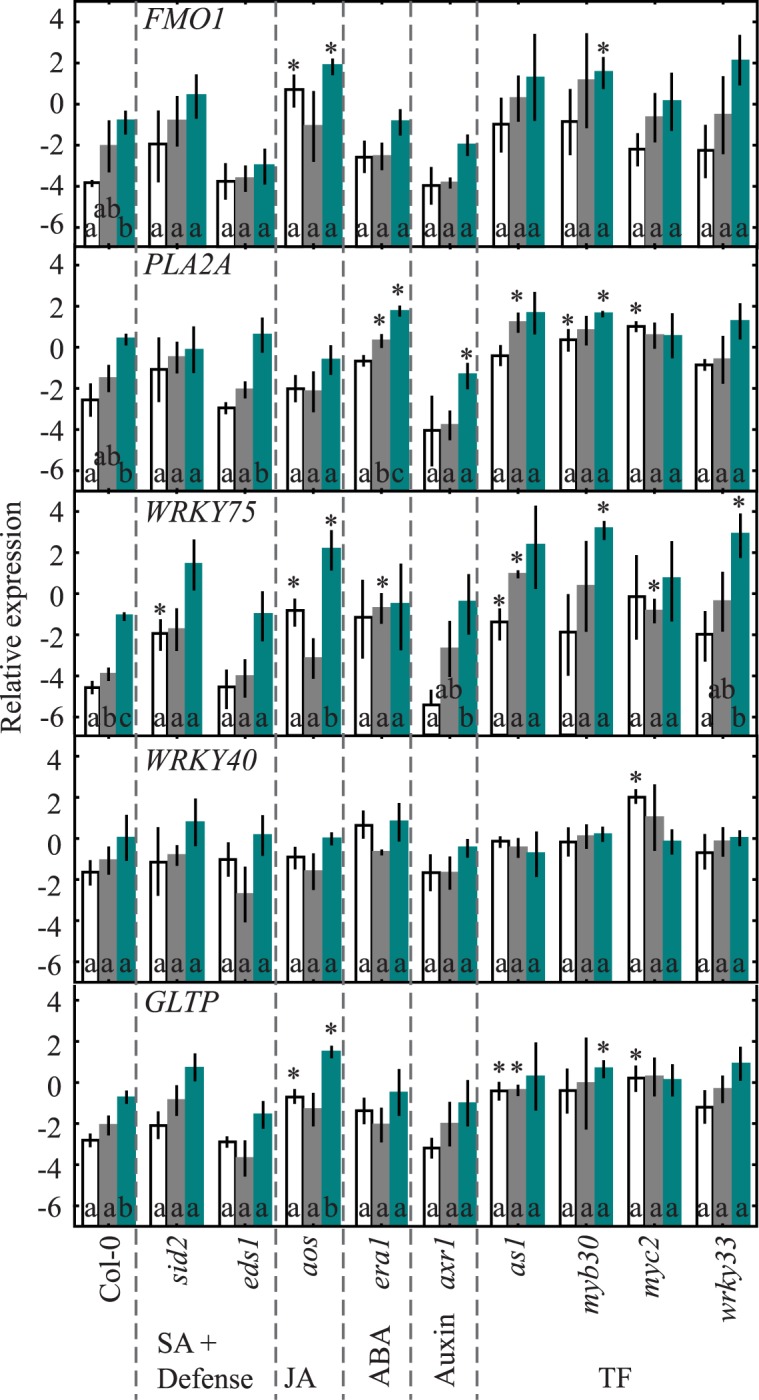
Marker gene expression in Col-0 and positive regulators of PCD in three leaf age classes. Mutants were divided into functional classes based on their primary function in defense signaling. Letters indicate differences between leaf age classes (p<0.05; n = 3) and asterisks differences relative to Col-0 at the corresponding leaf age. Leaves divided into age classes are: young (white boxes), mature (grey) and old (green). The values represent the mean (box) and standard error (bar).

A complementary approach to infer hormone or other regulation of the selected marker genes is to test their expression in mutants defective in hormone biosynthesis or signaling (Fig 1). We used mutants impaired in SA biosynthesis (*sid2*, which encodes ISOCHORISMATE SYNTHASE 1) or SA related signaling (*eds1*, *enhanced disease susceptibility1*), JA biosynthesis (*aos*, *allene oxide synthase*), altered ABA signaling (*era1*, *enhanced response to ABA1*), a regulatory component of auxin and JA receptors (*axr1*, *auxin resistant1*) and several transcription factors *as1*, *myb30*, *myc2*, and *wrky33* (Fig 4; Table 1). The transcription factor mutants were chosen based on their ability to partially or fully suppress cell death in the corresponding *cat2* double mutants [24].

Expression of *FMO1*, *PLA2A*, *WRKY75* and *GLTP* showed clear age related expression in Col-0, with maximum expression in old leaves (Fig 4). In contrast to the strong increased expression shown under various external hormone treatments (Fig 3), the influence of deficient hormone signaling in single mutants on age related expression was subtle. Significant changes in expression were observed in *aos* where *FMO1*, *WRKY75* and *GLTP* had increased transcript abundance in young and old leaves (Fig 4). Furthermore, *PLA2A*, *WRKY75* and *GLTP* expression was altered by lack of the TFs *as1*, *myb30* and *myc2* in various leaf age classes.

### Expression of Marker Genes in Young, Mature and Old *cat2* Leaves

Our previous analysis of cell death in 56 *cat2* double and triple mutants identified several mutations that lead to a reduction of cell death (*era1*, *eds1*, *sid2*, *axr1*, *as1*, *myc2*, *myb30*, *wrky33*, *dnd1*), indicating that the corresponding proteins are likely positive regulators of cell death (Fig 1, Table 1, [24]). To study the role of increased H_2_O_2_ (caused by *cat2* mutation) in age related gene expression the following mutants were included in the analysis: *cat2 sid2*, *cat2 eds1* and *cat2 sid2 eds1* (SA related mutants), *cat2 aos* (JA biosynthesis), *cat2 era1* (ABA signaling), *cat2 axr1* (auxin and JA signaling). In addition, *cat2* double mutants with transcription factors (TFs) that are positive regulators of cell death (AS1, MYC2, MYB30 and WRKY33; [24]) were included in the experiments to test the role of these TFs on the expression of the selected marker genes (Fig 1, Table 1).

**Fig 5:**
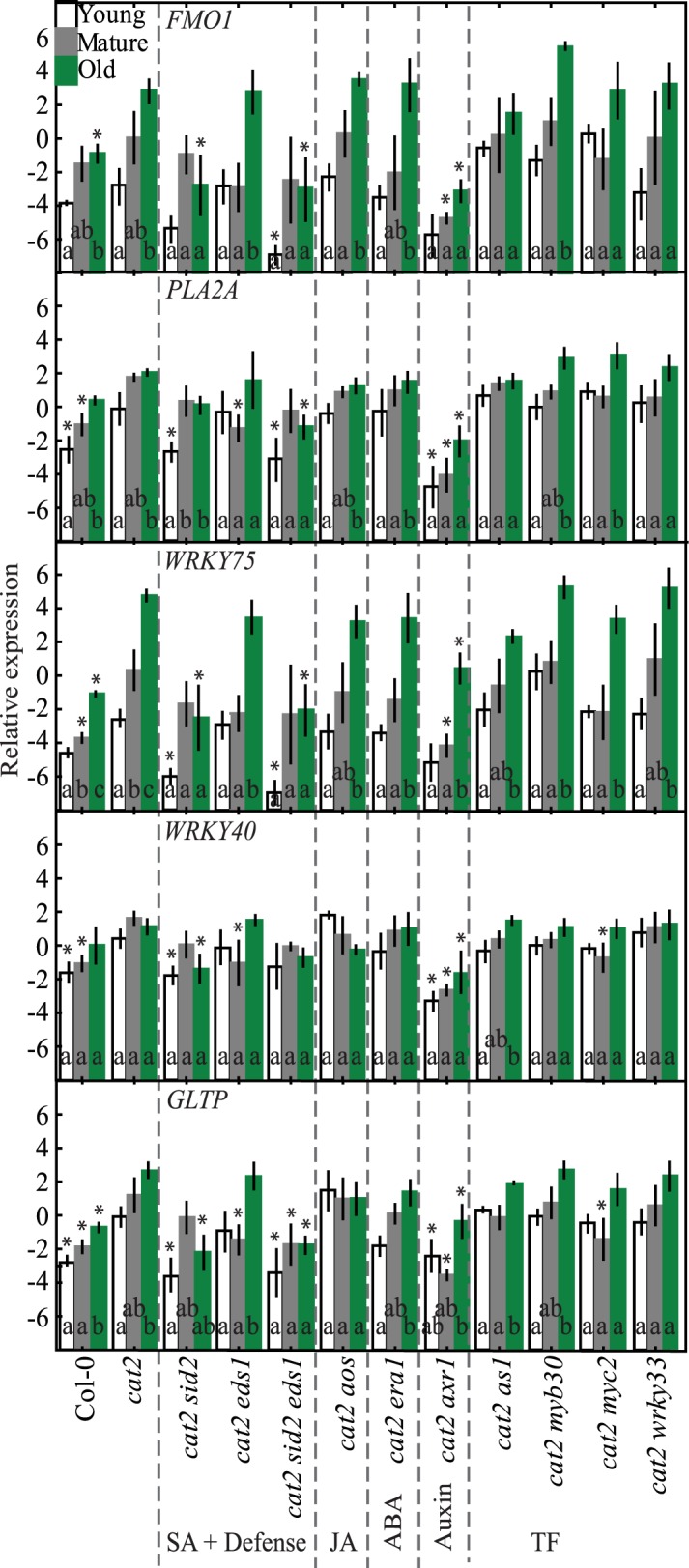
Marker gene expression in *cat2* and in double mutants defective in hormone signaling pathways or TFs. Letters indicate differences between leaf age classes (p<0.05; n = 3) and asteriks show significant differences relative to *cat2* at the corresponding leaf age. The values represent the mean (box) and standard error (bar).

Expression of *FMO1*, *WRKY75* and *GLTP* strongly increased in old *cat2* leaves compared to Col-0 (Fig 5). Impaired SA biosynthesis in *cat2 sid2* clearly decreased the expression of the stress marker genes, either in young or old leaves (Fig 5). Similarly, in *cat2 eds1* plants, the expression of *PLA2A*, *WRKY40* and *GLTP* was lower in mature leaves compared to *cat2*. Impairment of both SA biosynthesis and defense signaling via EDS1 in *cat2 sid2 eds1*, gave some subtle differences from *cat2 sid2*, e.g. a more pronounced decreased expression of the cell death regulator *PLA2A*.

Interestingly, and in contrast to previous studies describing the involvement of JA in plant defenses [41,42], the expression of *FMO1*, *PLA2A*, *WRKY75*, *WRKY40* and *GLTP* in *cat2 aos*, deficient in JA biosynthesis (and its precursor 12-oxo-phytodienoic acid), was not altered from the response in the single mutant *cat2* (Fig 5). Similarly, expression of defense genes was not altered in *cat2 era1*, indicating a minor role for ABA on the chosen PCD markers.

Auxin is essential during plant development and an important regulator of long-distance signaling [16,25,43]. In *cat2 axr1* expression of the five stress marker genes was reduced compared to *cat2* (Fig 5). Since AXR1 regulates the function of both auxin and JA signaling [44], the reduced expression of *FMO1*, *PLA2A*, *WRKY75*, *WRKY40* and *GLTP* in *cat2 axr1* could reflect impaired auxin signaling, JA signaling or both. However, given that marker gene expression *cat2 aos* was not affected by the *aos* mutation, this suggest that impairment of auxin signaling in *cat2 axr1* is more important for the low expression of the selected marker genes (Fig 5).

While several TFs regulate the extent of cell death in *cat2* [24], expression of the stress marker genes was not significantly different in *cat2* compared with double mutants defective in TFs, except for *cat2 myc2* (Fig 5). In this double mutant, the expression of *WRKY40* and *GLTP* was lower than in *cat2*.

### Expression of Marker Genes in Young, Mature and Old in *dnd1* Leaves

**Fig 6:**
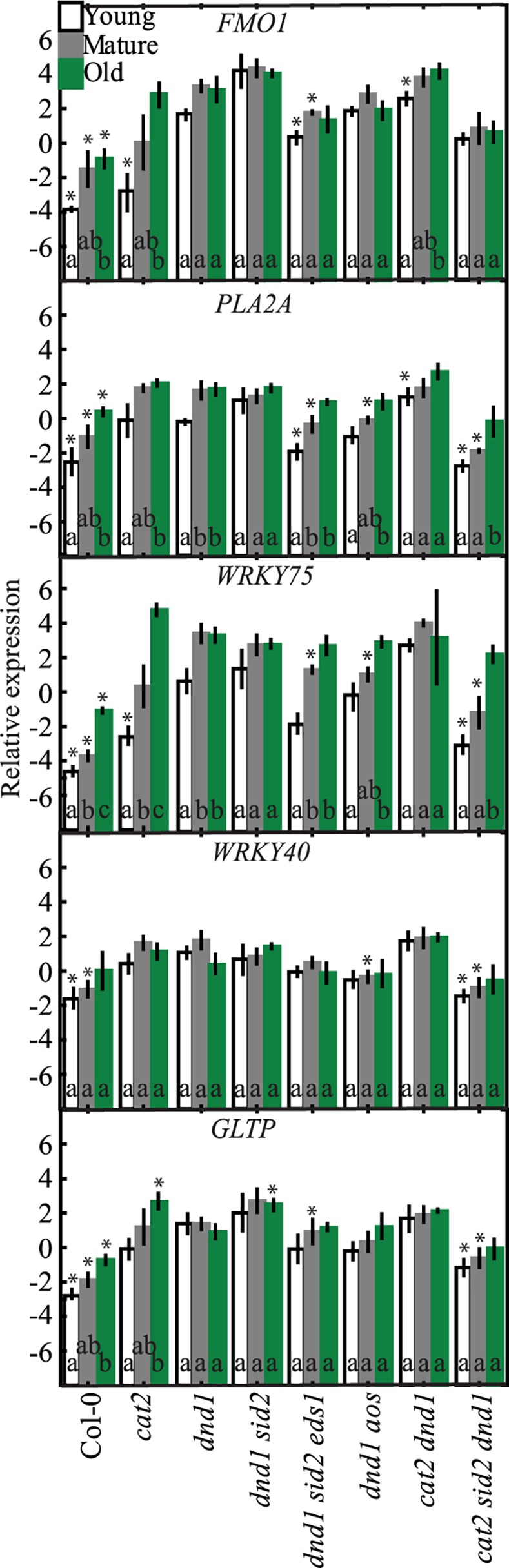
Marker gene expression in two LMMs *dnd1* and *cat2*. Letters indicate differences between leaf age classes (p<0.05; n = 3) and asteriks show significant differences relative to *dnd1* at the corresponding leaf age. The values represent the mean (box) and standard error (bar).

To extend the analysis of age and cell death related gene expression we analyzed a second LMM *dnd1* (Figs 1 and 6). As expected, all five marker genes had higher expression in *dnd1* than Col-0; furthermore, within the different age classes in *dnd1*, the expression of *PLA2A* and *WRKY75* increased with leaf age (Fig 6). The depletion of SA alone did not impair gene expression in *dnd1 sid2*, but *WRKY75*, *PLA2A* and *FMO1* expression was reduced to wild type level in *dnd1 sid2 eds1* young and mature leaves (Fig 6).

Although, the *aos* mutation did not influence age-related expression of stress marker genes in *cat2*, expression of *PLA2A*, *WRKY75* and *WRKY40* in mature leaves was significantly lower in *dnd1 aos* compared to *dnd1* (Fig 4). Interestingly, expression of defense genes in the double mutant *cat2 dnd1* was more similar to *dnd1* than *cat2*, indicating that the combination of two different mutations leading to spontaneous cell death did not lead to even higher defense gene expression. In the triple mutant *cat2 sid2 dnd1*, expression of four (*FMO1*, *PLA2A*, *WRKY75* and *GLTP*) of the five marker gene transcripts was significantly lower in mature and young leaves compared to *dnd1*, further emphasizing the important role for SA in defense gene expression (Fig 6).

### SA marker Gene Expression in *cat2 axr1*

**Fig 7:**
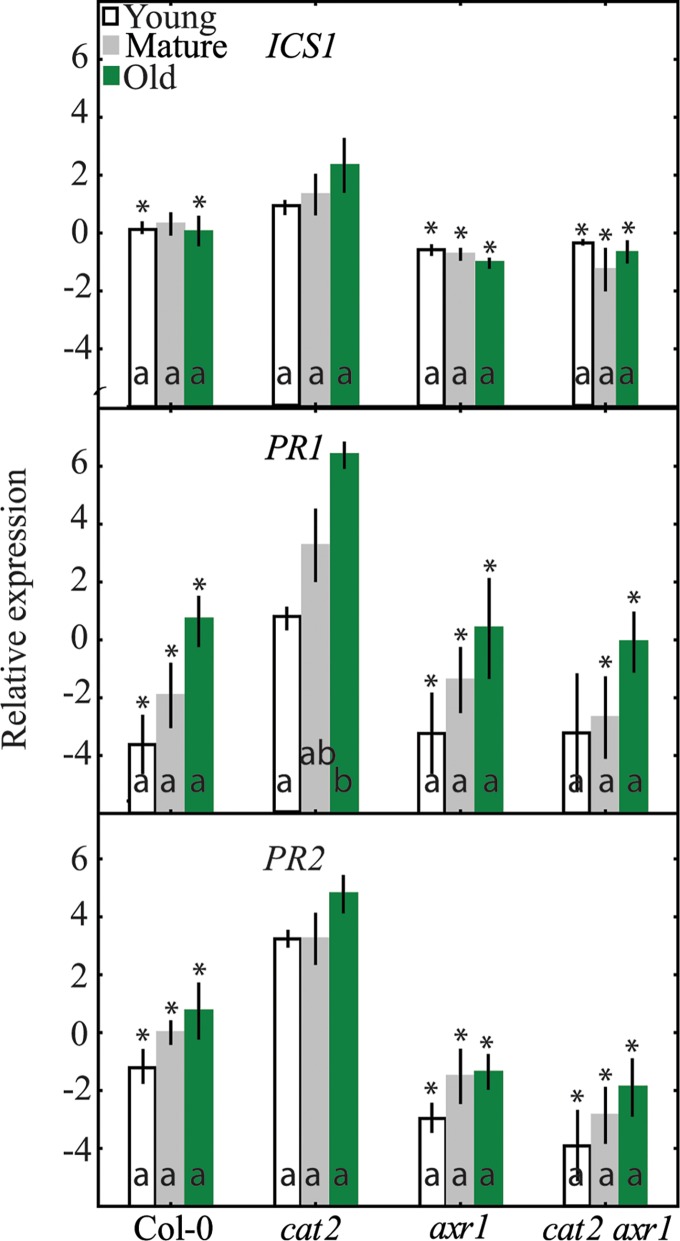
The expression of SA marker genes in Col-0, *cat2*, *axr1* and *cat2 axr1*. Letters indicate differences between leaf age classes (p<0.05; n = 3) and asterisks significant differences relative to *cat2* at the corresponding leaf age. The values represent the mean (box) and standard error (bar).

SA has a central role in execution of cell death in many LMMs, including *cat2* [22,24,45]. The molecular function of AXR1, regulation of SCF complexes that are involved in protein degradation [25], has been associated with auxin and JA responses [44]. The *cat2 axr1* double mutant displayed reduced cell death [24] and reduced marker gene expression (Fig 5), both of which were also seen in *cat2* double mutants with impaired SA biosynthesis and signaling [24,45]. This raised the question whether the *axr1* mutation could also directly affect SA signaling. We analyzed three marker genes for SA signaling; *ICS1*, *PR1* and *PR2* (Fig 7). All three genes were significantly increased in *cat2* in all leaf ages compared to wildtype, furthermore expression of *PR1* increased with age in *cat2* (Fig 7). Strikingly, no increased expression of SA marker genes was observed in *cat2 axr1*, raising the possibility that AXR1 regulation of SCF complexes also target the degradation of an essential component of SA signaling.

## Discussion

Systemic signaling is under intense study in plants. From a mechanistic perspective systemic signaling studies provide information on how cells and organs communicate with each other; from a practical perspective these studies aim to identify signals or even molecules that induce defense responses that could be of agricultural importance such as providing more rapid activation of defense after pathogen attack [46]. Here we used two different mutants that display increased defense gene expression and spontaneous cell death: *cat2* where cell death develops as a result of increased H_2_O_2_ production [12] and *dnd1* where cell death develops due to misregulated CYCLIC NUCLEOTIDE GATED CHANNEL 2 function and SA signaling [27]. The five marker genes (*FMO1*, *PLA2A*, *WRKY75*, *WRKY40* and *GLTP*) enabled evaluation of the role of leaf age on the expression of cell death related marker genes. In Col-0 and *cat2*, four of the genes (*FMO1*, *PLA2A*, *WRKY75* and *GLTP*) had lowest expression in young leaves, increasing in mature leaves and highest in old leaves. Similarly, *PLA2A* and *WRKY75* increased with leaf age in *dnd1*. Overall, this suggests that the youngest leaves have not yet entered into a strong defense or cell death program, and may instead be under regulation by a developmental program. Furthermore, the youngest leaves are likely to be sink leaves that receive their photosynthates from the mature and old leaves [47]. Especially in *cat2* this means that the photorespiration is lower in the youngest leaves and would not accumulate as high levels of H_2_O_2_ as the mature and old leaves. Consistent with this observation, when *cat2* is put into conditions of severe high light stress the youngest leaves do not develop cell death [47]. Similarly, in *dnd1*, spontaneous cell death is more prominent in the mature and old leaves [27]. High expression of defense genes in old leaves may be an adaptive response, since older leaves are more resistant to pathogens through e.g. accumulation of SA [48,49].

Previous studies have characterized the roles of *FMO1*, *PLA2A* and *WRKY75* in regulating defense signaling or cell death. For example, increased expression of *FMO1* is critical for execution of systemic acquired resistance (SAR) [50]. Furthermore, FMO1 also has a role in promotion of cell death [51]. High expression of *PLA2A* correlates with cell death, and transgenic overexpression of PLA2A enhances cell death [52,53]. WRKY75 controls crosstalk between SA/JA and ROS signals, all of which are required to activate defense regulation [54,55]. The *GLTP* gene in Arabidopsis has not been characterized. However, a related gene *ACCELERATED CELL DEATH 11* (*ACD11*) regulates cell death through transport of ceramide-1-phosphate and the *acd11* mutant exhibits runaway cell death [56–58]. The *acd11* mutant can be partially rescued by expression of a human *GLTP* [57], suggesting a cell death regulatory role also for Arabidopsis *GLTP*. Overall, the high expression of the PCD marker genes in this study and known lesion development in the oldest leaves of *cat2* [24,45] support a role for these genes in regulation of cell death.

Several signaling molecules are known to be involved in defense and systemic signaling and include SA, JA and ABA [15,32]. We selected *cat2* double mutants that have reduced cell death and evaluated the role of SA, JA, AXR1 and various TFs in the age regulated expression of cell death marker genes. While the TFs AS1, MYB30, MYC2 and WRKY33 regulated cell death in *cat2* [24], they had little influence on gene expression of the selected marker genes in *cat2* (Fig 5). Perhaps several TFs are acting together to regulate gene expression and knocking out multiple TFs would be required to see altered gene expression. SA is of central importance in the response to pathogens, in SAR and regulation of cell death [24,27,59]. Also the leaf age-dependent increase in expression of the marker genes in *cat2*, and to a lesser extent in *dnd1*, were reduced when SA biosynthesis was impaired through the *sid2* mutation or the combined *sid2 eds1* mutations (Figs 5 and 6). This is consistent with the protective role of SA in age-related resistance to pathogen infection [48].

The most striking reduction in marker gene expression was observed in *cat2 axr1* (Fig 5). AXR1 regulates the activity of multiple SCF complexes, where the auxin and JA insensitivity of the *axr1* mutant implicate the auxin receptor TIR1 and JA receptor COI1 as the major targets [25]. Since the JA deficient *cat2 aos* did not display altered expression compared to *cat2* (Fig 5), it is possible that the auxin insensitivity of *axr1* is more important for regulation of gene expression than the JA insensitivity. Previous studies related to *cat2* and oxidative stress have found that altered auxin signaling regulated the extent of PCD [24,60,61]. In large scale gene expression studies both SA and ROS lead to decreased expression of auxin related genes [62,63]. SA treatments showed that there is no immediate effect on auxin biosynthesis and instead SA suppresses auxin mediated genes mainly at the signaling level [63]. These results are consistent with the expression of the auxin signaling reporter gene *DR5* that was downregulated during oxidative stress [24,60,62]. Furthermore, expression of *GH3*.*3* (*Gretchen Hagen 3*.*3*) encoding an enzyme that conjugates auxin to amino acids have increased expression in systemically responding leaves after high light treatment [2]. However, subunits of the SCF complex are encoded by multiple genes, for example in Arabidopsis there are around 700 genes encoding the F-box protein, the subunit that determines substrate specificity [64]. The phenotypes of *axr1* have so far been explained by misregulated activity of the F-box proteins TIR1 (auxin receptor) and COI1 (JA receptor). However, other F-box proteins are also implicated in plant defense responses, including constitutive expresser of PR genes 30 (CPR30) that regulates some aspects of SA signaling [65]. Cell death in *cat2* is dependent on SA [24,45]. Low expression of cell death markers in *cat2 axr1* (Fig 5) as well as reduced cell death in this double mutant, could suggest that AXR1 also regulates a SCF complex that targets a component of SA signaling. We tested this idea directly using three SA markers genes (*ICS1*, *PR1*, *PR2*), which all had increased expression in *cat2* which was absent in *cat2 axr1* (Fig 7). Hence, in addition to its role in auxin and JA signaling, AXR1 may directly regulate SA signaling through misregulated F-box activity, and the *axr1* mutant would be deficient in SA signaling.

For simplicity, many studies on abiotic stress regulation of gene expression harvest entire seedlings, roots or rosettes. Given the clear difference in expression between young, mature and old leaves, more informative gene expression experiments should take advantage of tissue and cell specific assays [66]. Despite being one of the most studied hormones in relation to cell death, there is still a lack of information on exactly how SA regulates cell death. The identification of AXR1 as a regulator of cell death and SA gene expression signatures offers new opportunities to understand the regulation of cell death.

## Supporting Information

S1 Table Primer information and amplification efficiencies for qPCR analyses. (XLSX)

## References

[1] Coupe SA, Palmer BG, Lake JA, Overy SA, Oxborough K, Woodward FI, et al Systemic signalling of environmental cues in Arabidopsis leaves. J Exp Bot. 2006;57: 329–341. 10.1093/jxb/erj033 16330523

[2] Gordon MJ, Carmody M, Albrecht V, Pogson B. Systemic and Local Responses to Repeated HL Stress-Induced Retrograde Signaling in Arabidopsis. Front Plant Sci. 2013;3. 10.3389/fpls.2012.00303 PMC3547187 23335929

[3] Lake JA, Quick WP, Beerling DJ, Woodward FI. Plant development: Signals from mature to new leaves. Nature. 2001;411: 154–154. 10.1038/35075660 11346781

[4] Lake JA, Woodward FI, Quick WP. Long‐distance CO2 signalling in plants. J Exp Bot. 2002;53: 183–193. 11807121 10.1093/jexbot/53.367.183

[5] Foyer CH, Noctor G. Redox Regulation in Photosynthetic Organisms: Signaling, Acclimation, and Practical Implications. Antioxid Redox Signal. 2008;11: 861–905. 10.1089/ars.2008.2177 19239350

[6] Overmyer K, Brosché M, Kangasjärvi J. Reactive oxygen species and hormonal control of cell death. Trends Plant Sci. 2003;8: 335–342. 10.1016/S1360-1385(03)00135-3 12878018

[7] Mittler R, Vanderauwera S, Suzuki N, Miller G, Tognetti VB, Vandepoele K, et al ROS signaling: the new wave? Trends Plant Sci. 2011;16: 300–309. 10.1016/j.tplants.2011.03.007 21482172

[8] Suzuki N, Miller G, Morales J, Shulaev V, Torres MA, Mittler R. Respiratory burst oxidases: the engines of ROS signaling. Curr Opin Plant Biol. 2011;14: 691–699. 10.1016/j.pbi.2011.07.014 21862390

[9] Van Hautegem T, Waters AJ, Goodrich J, Nowack MK. Only in dying, life: programmed cell death during plant development. Trends Plant Sci. 2015;20: 102–113. 10.1016/j.tplants.2014.10.003 25457111

[10] Mullineaux PM, Baker NR. Oxidative Stress: Antagonistic Signaling for Acclimation or Cell Death?1. Plant Physiol. 2010;154: 521–525. 10.1104/pp.110.161406 20921177 PMC2949037

[11] Foyer CH, Noctor G. Oxidant and antioxidant signalling in plants: a re-evaluation of the concept of oxidative stress in a physiological context. Plant Cell Environ. 2005;28: 1056–1071.

[12] Queval G, Issakidis-Bourguet E, Hoeberichts FA, Vandorpe M, Gakière B, Vanacker H, et al Conditional oxidative stress responses in the Arabidopsis photorespiratory mutant cat2 demonstrate that redox state is a key modulator of daylength-dependent gene expression, and define photoperiod as a crucial factor in the regulation of H2O2-induced cell death. Plant J. 2007;52: 640–657. 10.1111/j.1365-313X.2007.03263.x 17877712

[13] Willekens H, Chamnongpol S, Davey M, Schraudner M, Langebartels C, Montagu MV, et al Catalase is a sink for H2O2 and is indispensable for stress defence in C3 plants. EMBO J. 1997;16: 4806–4816. 10.1093/emboj/16.16.4806 9305623 PMC1170116

[14] Allahverdiyeva Y, Battchikova N, Brosché M, Fujii H, Kangasjärvi S, Mulo P, et al Integration of photosynthesis, development and stress as an opportunity for plant biology. New Phytol. 2015; 208: 647–655. 10.1111/nph.13549 26174112

[15] Potters G, Pasternak TP, Guisez Y, Palme KJ, Jansen MAK. Stress-induced morphogenic responses: growing out of trouble? Trends Plant Sci. 2007;12: 98–105. 10.1016/j.tplants.2007.01.004 17287141

[16] Potters G, Pasternak TP, Guisez Y, Jansen M a. K. Different stresses, similar morphogenic responses: integrating a plethora of pathways. Plant Cell Environ. 2009;32: 158–169. 10.1111/j.1365-3040.2008.01908.x 19021890

[17] Gao Q-M, Zhu S, Kachroo P, Kachroo A. Signal regulators of systemic acquired resistance. Front Plant Sci. 2015;6. 10.3389/fpls.2015.00228 PMC4394658 25918514

[18] Pieterse CMJ, Zamioudis C, Berendsen RL, Weller DM, Wees SCMV, Bakker PAHM. Induced Systemic Resistance by Beneficial Microbes. Annu Rev Phytopathol. 2014;52: 347–375. 10.1146/annurev-phyto-082712-102340 24906124

[19] Mittler R, Blumwald E. The Roles of ROS and ABA in Systemic Acquired Acclimation. Plant Cell. 2015;27: 64–70. 10.1105/tpc.114.133090 25604442 PMC4330577

[20] Xia X-J, Zhou Y-H, Shi K, Zhou J, Foyer CH, Yu J-Q. Interplay between reactive oxygen species and hormones in the control of plant development and stress tolerance. J Exp Bot. 2015; 66: 2839–2856. 10.1093/jxb/erv089 25788732

[21] Ljung K, Nemhauser JL, Perata P. New mechanistic links between sugar and hormone signalling networks. Curr Opin Plant Biol. 2015;25: 130–137. 10.1016/j.pbi.2015.05.022 26037392

[22] Bruggeman Q, Raynaud C, Benhamed M, Delarue M. To die or not to die? Lessons from lesion mimic mutants. Front Plant Sci. 2015;6: 24 10.3389/fpls.2015.00024 25688254 PMC4311611

[23] Li Y, Chen L, Mu J, Zuo J. LESION SIMULATING DISEASE1 Interacts with Catalases to Regulate Hypersensitive Cell Death in Arabidopsis. Plant Physiol. 2013;163: 1059–1070. 10.1104/pp.113.225805 23958864 PMC3793025

[24] Kaurilind E, Xu E, Brosché M. A genetic framework for H2O2 induced cell death in Arabidopsis thaliana. BMC Genomics. 2015;16: 837 10.1186/s12864-015-1964-8 26493993 PMC4619244

[25] Dharmasiri N, Dharmasiri S, Weijers D, Karunarathna N, Jurgens G, Estelle M. AXL and AXR1 have redundant functions in RUB conjugation and growth and development in Arabidopsis. Plant J. 2007;52: 114–123. 10.1111/j.1365-313X.2007.03211.x 17655650

[26] Clough SJ, Fengler KA, Yu I -chin., Lippok B, Smith RK, Bent AF. The Arabidopsis dnd1 “defense, no death” gene encodes a mutated cyclic nucleotide-gated ion channel. Proc Natl Acad Sci. 2000;97: 9323–9328. 10.1073/pnas.150005697 10900264 PMC16866

[27] Xu E, Brosché M. Salicylic acid signaling inhibits apoplastic reactive oxygen species signaling. BMC Plant Biol. 2014;14: 155 10.1186/1471-2229-14-155 24898702 PMC4057906

[28] Ali R, Ma W, Lemtiri-Chlieh F, Tsaltas D, Leng Q, Bodman S von, et al Death Don’t Have No Mercy and Neither Does Calcium: Arabidopsis CYCLIC NUCLEOTIDE GATED CHANNEL2 and Innate Immunity. Plant Cell. 2007;19: 1081–1095. 10.1105/tpc.106.045096 17384171 PMC1867353

[29] Waszczak C, Kerchev PI, Mühlenbock P, Hoeberichts FA, Kelen KVD, Mhamdi A, et al SHORT-ROOT Deficiency Alleviates the Cell Death Phenotype of the Arabidopsis catalase2 Mutant under Photorespiration-Promoting Conditions. Plant Cell. 2016; tpc.00038.2016. 10.1105/tpc.16.00038 PMC5006698 27432873

[30] Khan M, Rozhon W, Poppenberger B. The Role of Hormones in the Aging of Plants—A Mini-Review. Gerontology. 2014;60: 49–55. 10.1159/000354334 24135638

[31] Hruz T, Laule O, Szabo G, Wessendorp F, Bleuler S, Oertle L, et al Genevestigator V3: A Reference Expression Database for the Meta-Analysis of Transcriptomes, Genevestigator V3: A Reference Expression Database for the Meta-Analysis of Transcriptomes. Adv Bioinforma Adv Bioinforma. 2008; e420747. 10.1155/2008/420747 PMC2777001 19956698

[32] Baxter A, Mittler R, Suzuki N. ROS as key players in plant stress signalling. J Exp Bot. 2014;65: 1229–1240. 10.1093/jxb/ert375 24253197

[33] Wildermuth MC, Dewdney J, Wu G, Ausubel FM. Isochorismate synthase is required to synthesize salicylic acid for plant defence. Nature. 2001;414: 562–565. 10.1038/35107108 11734859

[34] Falk A, Feys BJ, Frost LN, Jones JDG, Daniels MJ, Parker JE. EDS1, an essential component of R gene-mediated disease resistance in Arabidopsis has homology to eukaryotic lipases. Proc Natl Acad Sci. 1999;96: 3292–3297. 10077677 10.1073/pnas.96.6.3292 PMC15935

[35] Park J-H, Halitschke R, Kim HB, Baldwin IT, Feldmann KA, Feyereisen R. A knock-out mutation in allene oxide synthase results in male sterility and defective wound signal transduction in Arabidopsis due to a block in jasmonic acid biosynthesis. Plant J. 2002;31: 1–12. 12100478 10.1046/j.1365-313x.2002.01328.x

[36] Cutler S, Ghassemian M, Bonetta D, Cooney S, McCourt P. A Protein Farnesyl Transferase Involved in Abscisic Acid Signal Transduction in Arabidopsis. Science. 1996;273: 1239–1241. 8703061 10.1126/science.273.5279.1239

[37] Nurmberg PL, Knox KA, Yun B-W, Morris PC, Shafiei R, Hudson A, et al The developmental selector AS1 is an evolutionarily conserved regulator of the plant immune response. Proc Natl Acad Sci. 2007;104: 18795–18800. 10.1073/pnas.0705586104 18003921 PMC2141856

[38] Raffaele S, Vailleau F, Léger A, Joubès J, Miersch O, Huard C, et al A MYB Transcription Factor Regulates Very-Long-Chain Fatty Acid Biosynthesis for Activation of the Hypersensitive Cell Death Response in Arabidopsis. Plant Cell. 2008;20: 752–767. 10.1105/tpc.107.054858 18326828 PMC2329921

[39] Lorenzo O, Chico JM, Sánchez-Serrano JJ, Solano R. JASMONATE-INSENSITIVE1 Encodes a MYC Transcription Factor Essential to Discriminate between Different Jasmonate-Regulated Defense Responses in Arabidopsis. Plant Cell. 2004;16: 1938–1950. 10.1105/tpc.022319 15208388 PMC514172

[40] Zheng Z, Qamar SA, Chen Z, Mengiste T. Arabidopsis WRKY33 transcription factor is required for resistance to necrotrophic fungal pathogens. Plant J. 2006;48: 592–605. 10.1111/j.1365-313X.2006.02901.x 17059405

[41] Balbi V, Devoto A. Jasmonate signalling network in Arabidopsis thaliana: crucial regulatory nodes and new physiological scenarios. New Phytol. 2008;177: 301–318. 10.1111/j.1469-8137.2007.02292.x 18042205

[42] Han Y, Mhamdi A, Chaouch S, Noctor G. Regulation of basal and oxidative stress-triggered jasmonic acid-related gene expression by glutathione. Plant Cell Environ. 2013;36: 1135–1146. 10.1111/pce.12048 23210597

[43] Kazan K, Manners JM. Linking development to defense: auxin in plant–pathogen interactions. Trends Plant Sci. 2009;14: 373–382. 10.1016/j.tplants.2009.04.005 19559643

[44] Tiryaki I, Staswick PE. An Arabidopsis Mutant Defective in Jasmonate Response Is Allelic to the Auxin-Signaling Mutant axr1. Plant Physiol. 2002;130: 887–894. 10.1104/pp.005272 12376653 PMC166615

[45] Chaouch S, Queval G, Vanderauwera S, Mhamdi A, Vandorpe M, Langlois-Meurinne M, et al Peroxisomal Hydrogen Peroxide Is Coupled to Biotic Defense Responses by ISOCHORISMATE SYNTHASE1 in a Daylength-Related Manner. Plant Physiol. 2010;153: 1692–1705. 10.1104/pp.110.153957 20543092 PMC2923881

[46] Conrath U, Beckers GJM, Langenbach CJG, Jaskiewicz MR. Priming for Enhanced Defense. Annu Rev Phytopathol. 2015;53: 97–119. 10.1146/annurev-phyto-080614-120132 26070330

[47] Kerchev PI, Waszczak C, Lewandowska A, Willems P, Shapiguzov A, Li Z, et al Lack of GLYCOLATE OXIDASE 1, but not GLYCOLATE OXIDASE 2, attenuates the photorespiratory phenotype of CATALASE2-deficient Arabidopsis. Plant Physiol. 2016; pp.00359.2016. 10.1104/pp.16.00359 PMC4936566 27225899

[48] Carella P, Wilson DC, Cameron RK. Some things get better with age: differences in salicylic acid accumulation and defense signaling in young and mature Arabidopsis. Plant-Microbe Interact. 2015;5: 775. 10.3389/fpls.2014.00775 PMC4288333 25620972

[49] Kus JV, Zaton K, Sarkar R, Cameron RK. Age-Related Resistance in Arabidopsis Is a Developmentally Regulated Defense Response to Pseudomonas syringae. Plant Cell. 2002;14: 479–490. 10.1105/tpc.010481 11884688 PMC152926

[50] Mishina TE, Zeier J. The Arabidopsis Flavin-Dependent Monooxygenase FMO1 Is an Essential Component of Biologically Induced Systemic Acquired Resistance. Plant Physiol. 2006;141: 1666–1675. 10.1104/pp.106.081257 16778014 PMC1533925

[51] Bartsch M, Gobbato E, Bednarek P, Debey S, Schultze JL, Bautor J, et al Salicylic Acid–Independent ENHANCED DISEASE SUSCEPTIBILITY1 Signaling in Arabidopsis Immunity and Cell Death Is Regulated by the Monooxygenase FMO1 and the Nudix Hydrolase NUDT7. Plant Cell. 2006;18: 1038–1051. 10.1105/tpc.105.039982 16531493 PMC1425861

[52] Camera SL, Balagué C, Göbel C, Geoffroy P, Legrand M, Feussner I, et al The Arabidopsis Patatin-Like Protein 2 (PLP2) Plays an Essential Role in Cell Death Execution and Differentially Affects Biosynthesis of Oxylipins and Resistance to Pathogens. Mol Plant Microbe Interact. 2009;22: 469–481. 10.1094/MPMI-22-4-0469 19271961

[53] La Camera S, Geoffroy P, Samaha H, Ndiaye A, Rahim G, Legrand M, et al A pathogen-inducible patatin-like lipid acyl hydrolase facilitates fungal and bacterial host colonization in Arabidopsis. Plant J. 2005;44: 810–825. 10.1111/j.1365-313X.2005.02578.x 16297072

[54] Encinas-Villarejo S, Maldonado AM, Amil-Ruiz F, Santos B de los, Romero F, Pliego-Alfaro F, et al Evidence for a positive regulatory role of strawberry (Fragaria×ananassa) Fa WRKY1 and Arabidopsis At WRKY75 proteins in resistance. J Exp Bot. 2009;60: 3043–3065. 10.1093/jxb/erp152 19470657

[55] Schmiesing A, Emonet A, Gouhier-Darimont C, Reymond P. Arabidopsis MYC Transcription Factors Are the Target of Hormonal SA/JA Crosstalk in Response to Pieris brassicae Egg Extract. Plant Physiol. 2016; pp.00031.2016. 10.1104/pp.16.00031 PMC4825139 26884488

[56] Inzé A, Vanderauwera S, Hoeberichts FA, Vandorpe M, Van Gaever T, Van Breusegem F. A subcellular localization compendium of hydrogen peroxide-induced proteins. Plant Cell Environ. 2012;35: 308–320. 10.1111/j.1365-3040.2011.02323.x 21443605

[57] Petersen NHT, McKinney LV, Pike H, Hofius D, Zakaria A, Brodersen P, et al Human GLTP and mutant forms of ACD11 suppress cell death in the Arabidopsis acd11 mutant. FEBS J. 2008;275: 4378–4388. 10.1111/j.1742-4658.2008.06584.x 18657186 PMC2585820

[58] Simanshu DK, Zhai X, Munch D, Hofius D, Markham JE, Bielawski J, et al Arabidopsis Accelerated Cell Death 11, ACD11, Is a Ceramide-1-Phosphate Transfer Protein and Intermediary Regulator of Phytoceramide Levels. Cell Rep. 2014;6: 388–399. 10.1016/j.celrep.2013.12.023 24412362 PMC3931444

[59] Lorrain S, Vailleau F, Balagué C, Roby D. Lesion mimic mutants: keys for deciphering cell death and defense pathways in plants? Trends Plant Sci. 2003;8: 263–271. 10.1016/S1360-1385(03)00108-0 12818660

[60] Gao X, Yuan H-M, Hu Y-Q, Li J, Lu Y-T. Mutation of Arabidopsis CATALASE2 results in hyponastic leaves by changes of auxin levels. Plant Cell Environ. 2014;37: 175–188. 10.1111/pce.12144 23738953

[61] Kerchev P, Mühlenbock P, Denecker J, Morreel K, Hoeberichts FA, Van Der Kelen K, et al Activation of auxin signalling counteracts photorespiratory H2O2-dependent cell death. Plant Cell Environ. 2015;38: 253–265. 10.1111/pce.12250 26317137

[62] Blomster T, Salojärvi J, Sipari N, Brosché M, Ahlfors R, Keinänen M, et al Apoplastic Reactive Oxygen Species Transiently Decrease Auxin Signaling and Cause Stress-Induced Morphogenic Response in Arabidopsis. Plant Physiol. 2011;157: 1866–1883. 10.1104/pp.111.181883 22007024 PMC3327221

[63] Wang D, Pajerowska-Mukhtar K, Culler AH, Dong X. Salicylic Acid Inhibits Pathogen Growth in Plants through Repression of the Auxin Signaling Pathway. Curr Biol. 2007;17: 1784–1790. 10.1016/j.cub.2007.09.025 17919906

[64] Sadanandom A, Bailey M, Ewan R, Lee J, Nelis S. The ubiquitin–proteasome system: central modifier of plant signalling. New Phytol. 2012;196: 13–28. 10.1111/j.1469-8137.2012.04266.x 22897362

[65] Gou M, Su N, Zheng J, Huai J, Wu G, Zhao J, et al An F-box gene, CPR30, functions as a negative regulator of the defense response in Arabidopsis. Plant J. 2009;60: 757–770. 10.1111/j.1365-313X.2009.03995.x 19682297

[66] Bailey-Serres J. Microgenomics: Genome-Scale, Cell-Specific Monitoring of Multiple Gene Regulation Tiers. Annu Rev Plant Biol. 2013;64: 293–325. 10.1146/annurev-arplant-050312-120035 23451787

